# Norovirus outbreaks in long-term care facilities in Catalonia from 2017 to 2018

**DOI:** 10.1038/s41598-021-02348-2

**Published:** 2021-12-01

**Authors:** Ignacio Parrón, Irene Barrabeig, Miquel Alseda, Cristina Rius, Thais Cornejo-Sánchez, Mireia Jané, Cristina Pérez, Susana Guix, Àngela Domínguez, the Working Group for the Study of Outbreaks of Acute Gastroenteritis in Catalonia, Cristina Pérez, Josep Álvarez, Irene Barrabeig, Maria Rosa Sala, Anna Isabel Belver, Ariadna Rovira, Ignacio Parrón, Lorena Coronas, Miquel Alsedà, Pere Godoy, Anna de Andres, Javier de Benito, Esteve Camprubí, Montse Cunillé, M. Lluïsa Forns, Antonio Moreno-Martínez, Efrén Razquín, Sara Sabaté, Mercé de Simón, Cristina Rius, Àngela Domínguez, Núria Soldevila, Rosa Bartolomé, Thais Cornejo-Sánchez, Mireia Jané, Ana Martínez, Núria Torner, Conchita Izquierdo, Rosa Maria Vileu, Susana Guix, Neus Camps, Maria Sabaté, Sofia Minguell, Monica Carol

**Affiliations:** 1 Sub-Direcció Regional a Barcelona del Departament de Salut, Barcelona, Spain; 2 Departament de Medicina, Universitat de Barcelona, Barcelona, Spain; 3 CIBER Epidemiologia y Salud Pública, Instituto de Salud Carlos III, Madrid, Spain; 4 Sub-Direcció Regional a Lleida del Departament de Salut, Lleida, Spain; 5 Agència de Salut Pública de Barcelona, Barcelona, Spain; 6 Departament de Microbiologia, Vall d’Hebrón Hospital, Barcelona, Spain; 7 Sub-Direcció General de Vigilància i Resposta a Emergències de Salut Pública, Barcelona, Spain; 8 Departament de Genètica Microbiologia i Estadística, Grup de Virus Entèrics, Universitat de Barcelona, Barcelona, Spain; 9 Institut de Recerca en Nutrició i Seguretat Alimentària (INSA-UB), Universitat de Barcelona, Santa Coloma de Gramenet, Spain; 10 Sub-Direcció Regional a Girona del Departament de Salut, Girona, Spain; 11 Sub-Direcció Regional a Tarragona del Departament de Salut, Tarragona, Spain; 12 Sub-Direcció Regional a Catalunya Central del Departament de Salut, Manresa, Spain

**Keywords:** Epidemiology, Viral epidemiology, Gastroenteritis, Geriatrics

## Abstract

Norovirus is the leading cause of outbreaks of acute viral gastroenteritis. We carried out this study to investigate outbreaks in long-term care facilities reported in 2017 and 2018 in Catalonia (Spain). The characteristics of the centers, exposed persons and the genogroups responsible were analyzed. Viral loads were estimated. The attack rate (AR) of the outbreaks studied, and the rate ratio (RR) and the odds ratio (OR) and their 95% confidence intervals as measures of association were calculated. The mean cycle thresholds were compared using the t-test for independent means. We included 30 outbreaks (4631 exposed people). The global AR was 25.93%. The RR of residents vs. staff was 2.28 (95% CI 2.0–2.6). The RR between AR in residents with total or severe dependence vs. residents with moderate, low or no-dependence was 1.23 (95% CI 1.05–1.45). The AR were higher in smaller centers than in larger ones (38.47% vs. 19.25% and RR 2; 95% CI 1.82–2.2). GII was responsible for 70% of outbreaks. No association was found between the genogroup and presenting symptoms (OR 0.96; 95% CI 0.41–2.26). Viral loads were higher in symptomatic than in asymptomatic patients (*p* = 0.001).

## Introduction

Norovirus, an RNA virus of the *Caliciviridae* family with 10 genogroups, of which genogroup I (GI), genogroup II (GII) and genogroup IV (GIV) are human pathogens^1^, usually produces symptoms of nausea, vomiting and diarrhea, with a self-limiting evolution of 48–72 h^2^.

Norovirus is estimated to be responsible for 20% of cases of all-cause diarrhea worldwide^3^ and may cause up to 90% of outbreaks of acute gastroenteritis (AGE) of viral etiology^4^.

Inns et al., in a review of norovirus reports worldwide between 1995 and 2015, found an incidence of up to 60 cases per 1000 person-years and a hospitalization rate of up to 1.04 per 1000 person-years^5^. Kreidieh et al.^6^ in a similar study in the Middle East and North Africa between 2000 and 2015 found that between 0.82% and 36.84% of AGE outbreaks in children aged < 5 years treated in hospital emergency rooms were caused by norovirus.

Of the more than 1000 outbreaks of AGE reported annually in 2009 and 2010 in the United States, norovirus was confirmed as the etiological agent in 86%, and 90% of norovirus-associated deaths occurred in people aged ≥ 65 years^7^.

In long-term care facilities (LTCF), the attack rate of AGE outbreaks due to norovirus varies between 3% and 45%, with a case fatality rate ranging from 0.3% to 1.6%^8^. In these institutions, norovirus is the second leading cause of outbreaks, after the influenza virus^9^. In England, an incidence of 30 outbreaks per 100 LTCF per year was reported in 2014–2016^10^. In France in 2011, more than 70% of AGE outbreaks in LTCF were due to norovirus^11^ and in the United States > 60% of norovirus outbreaks between 2009 and 2013 occurred in LTCF^12^. Although norovirus infection is usually mild, it may be more severe in older people. In developed countries, norovirus is responsible for between 10% and 20% of hospitalizations due to AGE in residents of LTCF and between 10% and 15% of deaths^8^.

GII is the most frequently identified genogroup of norovirus in outbreaks^13^. In Spain, this genogroup has also been the most prevalent in recent years^14^.

Asymptomatic affected people may contribute to the transmission of norovirus^15^ and to a longer duration of outbreaks, which has important repercussions for disease control.

Symptomatic persons have a higher viral load than asymptomatic ones^16^, but no value has been established to predict the level of shedding associated with clinical manifestations^17^.

In symptomatic patients, it has not been possible to associate the duration of symptoms with the viral load, although the duration of viral shedding has been shown to be longer in those with a higher load and in older people^18,19^.

In patients with AGE, the viral load has been shown to be higher when symptoms are due to GII rather than GI, and the higher load of GII, which has been linked to increased ease of transmission^20^, has also been observed in patients co-infected with GI and GII^16,18,21,22^.

The objective of this study was to investigate attack rates in AGE outbreaks due to norovirus that occurred in LTCF and their association with the type of exposed person, the size of the center, the mode of transmission, the genogroup involved, and the viral load.

## Materials and methods

A prospective study of outbreaks of AGE due to norovirus in LTCF reported between January 2017 and December 2018 was carried out in Catalonia, a region in Northeast Spain with a population of 7,496,276 in January 2017, of which 18.6% were aged ≥ 65 years^23^ and 59,635 were residents of LTCF^24^.

Outbreaks of any etiology must be reported to the Public Health Agency of Catalonia, which studies the causes and establishes control measures^25^.

AGE was defined as sudden-onset diarrhea that may be accompanied by fever, nausea, vomiting, or abdominal pain. The involvement of ≥ 2 people with a common exposure (or possible person-to-person transmission) was considered as an outbreak of AGE. A confirmed outbreak of norovirus was defined as the identification of norovirus in stool samples by real-time semiquantitative reverse transcription polymerase chain reaction (RTqPCR).

Two periods were defined: the warm months, lasting from April through September, and the cool months, lasting from October through March of the following year.

### Data collection

All norovirus outbreaks occurring in LTCF confirmed by RTqPCR reported between January 2017 and December 2018 were included. The numbers of residents and staff (affected and unaffected), the capacity of the center and whether transmission was person-to-person or by a common vehicle were collected.

A survey was designed for exposed persons including sociodemographic data, the degree of dependence (estimated using the Barthel index^26^), the history of heart disease, diabetes mellitus, dementia, immunodeficiency and chronic obstructive pulmonary disease, the date and time of symptom onset, the symptoms presented, hospitalization and death. For staff, information on the type of work was collected.

### Stool sample analysis

Stool samples were collected to identify the cause of the outbreak. Norovirus was tested for using RTqPCR and identifying the genogroup detected. The semi-quantitative value given by the RTqPCR cycle of quantification (Cq) was used to measure the viral load in samples positive for norovirus. Stool samples were obtained from symptomatic and asymptomatic staff and residents and, in outbreaks where foodborne transmission was suspected, from food handlers.

The analyses were made in the microbiology laboratories of the Vall Hebrón University Hospital and the Public Health Agency of Barcelona. Allplex GI-Virus Assay, Seegene Inc, was used to detect norovirus GI and GII. Samples positive for norovirus were genotyped using the primers described by Kojima et al.^27^. After the sequences were obtained, the Norovirus Typing Tool Version 2.0 (https://www.rivm.nl/mpf/typingtool/norovirus/) was used to obtain the genotype.

### Statistical analysis

The attack rate (AR) by age group, sex, relation with the center (residents or staff), mode of transmission (person-to-person or foodborne), degree of dependency and, in staff, type of work activity were calculated. The rate ratio (RR) and 95% confidence intervals (CI) were calculated to estimate the risk of being affected globally and separately for sex, mode of transmission, the capacity of the center (< 100 residents or ≥ 100 residents) and the level of dependence (residents with total or severe dependence vs. residents with moderate, low or no-dependence).

To assess the seasonality, we used a one-tailed Z-test to compare the proportion of outbreaks in the cool months (October to March) with a theoretical value of 50%. The correlation between the average temperature of each month in Barcelona^28^ and the number of outbreaks in the month was estimated using Pearson’s correlation coefficient and the *p* value with a t-Student test.

A one-tailed Fisher’s exact test was used to compare the proportion of residents affected with specific underlying diseases (heart disease, diabetes mellitus, dementia, immunodeficiency and chronic obstructive pulmonary disease) with the proportion of residents without these underlying diseases. This test was also used to compare the proportion of residents and staff members who needed medical care. The association between genogroup and the presence of symptoms was assessed using the odds ratio (OR) and its 95% CI.

The mean Cq, as an approximation to the viral load, was compared in symptomatic and asymptomatic infected persons (in staff, residents and all attenders) using the t-test for independent means. The Student’s t-test and its 95% CI was used to calculate the degree of significance of the difference between means. A Hartley's F_max_ test for variance homogeneity was used previously and if the *p* value of the F_max_ statistic was > 0.05, the t-test was based on equal variance; otherwise, the t-test was based on unequal variance.

Data collection and management was made using the MS-Office 2013 Access 12.0 database and the statistical analysis using the PASW Statistics 18.0.2 statistical package and Epi Info for Windows 7.2.

### Ethics declarations and informed consent statement

The study was conducted according to the guidelines of the Declaration of Helsinki, regulations of the Public Health Agency of Catalonia and ethical protocols established. The study was approved by the University of Barcelona Bioethics Commission (ethics approval number IRB00003099) on April 12, 2016.

The authors declare that the Bioethics Committee of University of Barcelona approved the waiver for informed consent. All data used in the analysis were collected during routine public health surveillance activities as part of the legislated mandate of the Health Department of Catalonia, which is officially authorized to receive, treat and temporarily store personal data in the case of infectious disease. All data were fully anonymized. All study activities formed part of the public health surveillance tasks. The law regulates these activities and informed consent should not be necessary.

## Results

### Reported outbreaks

During the study period, 213 AGE outbreaks were reported to the Public Health Agency of Catalonia; 40 (18.78%) occurred in LTCF and norovirus was identified as the causal agent in 75% (30/40).

The transmission mode was person-to-person in 27 of the 30 outbreaks in LTCF and foodborne in the remaining three, although there was also subsequent person-to-person transmission in one foodborne outbreak. In these three foodborne outbreaks, four kitchen workers were affected (3 cooks and 1 kitchen assistant).

A total of 4631 persons were exposed and 1201 of these were affected (AR 25.93%); 3,034 exposed persons were LTCF residents, of whom 976 were affected (AR 32.17%). Of the 1597 exposed staff members, 225 were affected (AR 14.09%). The RR for residents vs. staff members was 2.28 (95% CI 2.0–2.61).

In person-to-person transmission outbreaks, 4170 persons were exposed and 1048 were affected (AR 25.13%). Among residents, the AR was 32.48% (2768 exposed and 899 affected) and among staff members it was 10.63% (1402 exposed and 149 affected) (RR 3.06 95% CI 2.60–3.59).

In foodborne outbreaks, the overall AR was 33.19% (461 exposed and 153 affected), and it was 28.95% (266 exposed and 77 affected) among residents and 38.97% (195 exposed and 76 affected) among staff (RR 0.74; 95% CI 0.57–0.96).

**Figure 1:**
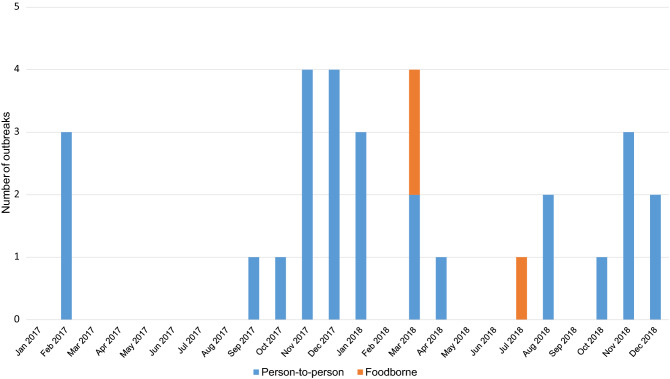
Number of acute gastroenteritis outbreaks due to norovirus in long-term care facilities according to the month of onset and mode of transmission.

Twenty-five (83.33%) outbreaks occurred in the cool months and five (16.67%) in the warm months. There was a significantly higher proportion of outbreaks in the cool months (z-test = 3.61, *p* < 0.001). In addition, a Pearson’s correlation coefficient of − 0.63 (*p* = 0.03) between the average temperature and the number of outbreaks per month was observed. Of the 27 outbreaks with person-to-person transmission, 13 (48.15%) occurred during the colder months (December to March) (Fig. 1).

Of the centers where outbreaks occurred, 13 had a capacity of ≥ 100 residents and the AR was 19.26%. The remaining 17 centers had a capacity of < 100 residents and the AR was 38.47%. The RR, both globally and for staff or residents, indicated an increased risk of being affected in smaller centers (Table 1).

**Table 1:** Attack rates and rate ratio (RR) according to the capacity of the facility (separately for residents and staff and globally).

	Centers with < 100 residents			Centers with ≥ 100 residents			RR (95% CI)
	Affected	Unaffected	Attack rate	Affected	Unaffected	Attack rate	
Residents	508	616	45.2	468	1442	24.5	1.85 (1.67–2.04)
Staff	111	374	22.89	114	998	10.25	2.23 (1.76–2.83)
Total exposed	619	990	38.47	582	2440	19.26	2 (1.82–2.2)

### Results of the survey in exposed and affected persons in LTCF

A total of 495 exposed persons (365 residents and 130 staff) were interviewed, of whom 106 (21.41%) were male and 389 (78.59%) female. The average age was 86.20 years (SD 8.87) in residents and 40.14 years (SD 13.44) in staff. Nine residents aged < 65 years were affected (AR 88.89%), 64 aged 65–74 years (AR 84.21%), 158 aged 85–94 years (AR 82.29%) and 36 aged 95–105 years (AR 83.72%). In staff members, there were 38 affected persons aged 18–39 years (AR 65.52%) and 34 aged 40–65 years (AR 53.97%). The age was unknown in 20 residents and 9 staff members.

Of the total exposed people interviewed, 371 persons were affected (295 residents and 76 staff members) and 124 unaffected. Nine residents and 13 staff members needed medical care (3.05% of affected residents and 17.11% of affected staff members), with the difference being significant (*p* < 0.001). Four affected persons (1.08% of all affected people) were hospitalized: 3 residents (1.02% of residents affected) and 1 staff member (1.32% of staff members affected). The difference was not significant (*p* = 0.60). No deaths were recorded.

The AR was 72.64% in males (106 exposed and 77 affected) and 75.58% in females (389 exposed, 294 affected) (RR 0.96; 95% CI 0.84–1.09).

The AR was 76.55% in person-to-person outbreaks (388 exposed, 297 affected) and 69.16% (107 exposed, 74 affected) in foodborne outbreaks (RR 1.11; 95% CI 0.96–1.27).

Information on underlying diseases was obtained in 305 (83.56%) residents of whom 255 were affected. The proportion of residents with and without a specific underlying disease who were affected were compared: for heart disease these proportions were 86.33% and 81.33% (*p* = 0.15); for diabetes mellitus 82.89% and 64.21% (*p* = 0.49); for dementia 81.03% and 84.21% (*p* = 0.34); for immunodeficiency 81.82% and 83.67% (*p* = 0.56); for chronic obstructive pulmonary disease 76.74% and 84.73% (*p* = 0.14). The degree of dependence (measured by the Barthel index) was obtained in 236 residents (188 affected and 48 unaffected). The AR was 85.16% in residents with total or severe dependence and 69.13% in residents with moderate, low or no-dependence (RR 1.23; 95% CI 1.05–1.45) (Table 2).

**Table 2:** Degree of dependency (Barthel index) in affected and unaffected residents, attack rates and rate ratio (RR) in residents with total or severe dependence versus residents with moderate, low or no-dependence.

Degree of dependency	Affected residents	Unaffected residents	Attack rate (%)	RR (95% CI)
Total dependency (0–20)	45	13	77.59	
Severe dependency (21–60)	87	10	89.69	
Moderate dependency (61–90)	48	22	68.57	
Low dependency (91–99)	5	2	71.43	
Independent (100)	3	1	75	
Total + severe	132	23	85.16	1.23 (1.05–1.45)
Moderate + low + No dependence	56	25	69.13	1

Information on occupation was obtained for 123 staff members: 58 were maintenance personnel or kitchen staff (of whom 26 were affected) and 65 were healthcare staff or caregivers (of whom 49 were affected); in 7 staff members this information was not available. Kitchen staff and maintenance personnel had a lower risk of being affected than healthcare staff and caregivers (RR 0.59; 95% CI 0.43–0.82) (Table 3).

**Table 3:** Attack rates and rate ratio (RR) in long-term care facilities staff according to occupation.

Occupation	Affected	Unaffected	Attack rate (%)	RR (95% CI)
Kitchen staff and service personnel	26	32	44.83	0.59 (0.43–0.82)
Health staff and caregivers	49	16	75.38	1
Total	75	48	60.98	

Of the 30 outbreaks studied, 5 were due to GI (16.67%), 21 to GII (70%) and the remaining 4 (13.33%) were due to mixed infection by GI and GII. We identified 35 genotypes: GII.4 was identified in 13 outbreaks, GII.17 in five, GI.6 in three, and GI.3, GI.4, GII.2 and GII.P16 in two outbreaks. The remaining genotypes (GI.2, GI.5, GI.P4, GI.P5, GII.P7, GII.P17 and GII.P31) were identified in one outbreak.

A total of 425 stool samples were collected and norovirus was identified by RTqPCR in 256. GI was identified in 53 samples (20.70%) and GII in 198 samples (77.34%). In 5 samples (1.95%) coinfection with GI and GII (2 symptomatic and 3 asymptomatic) was identified. The remaining 169 samples were negative for norovirus.

Table 4 shows the genogroup detected in symptomatic and asymptomatic infected LTCF staff and residents. No association was found between the genogroup and presenting symptoms (OR 0.96; 95% CI 0.41–2.26), indicating that the proportion of asymptomatic infections was similar for both genogroups.

**Table 4:** Norovirus genogroup in symptomatic and asymptomatic infected long-term care facilities staff and residents.

Genogroup	Symptomatic	Asymptomatic	Total	OR (95% CI)
GI	45	8	53	0.96 (0.41–2.26)
GII	169	29	198	1

With respect to the viral load in symptomatic and asymptomatic persons (Table 5), the difference between the means of the Cq was − 3.35 (95% CI − 5.34 to − 1.35), with a greater viral load found in symptomatic than in asymptomatic persons (*p* = 0.001).

**Table 5:** Quantification cycle (Cq) values in symptomatic and asymptomatic infected staff and residents of long-term care facilities.

	Symptomatic	Mean (SD)	Positive samples*	Difference between means of Cq (95% CI)	*p* Value
Staff	Yes	28.11 (5.54)	46	− 1.85 (− 4.87 to 1.17)	0.225
	No	29.96 (5.15)	18		
Residents	Yes	24.96 (5.87)	164	− 3.19 (− 5.82 to − 0.56)	0.018
	No	28.15 (5.84)	22		
All persons	Yes	25.65 (5.93)	210	− 3.35 (− 5.34 to − 1.35)	0.001
	No	29.00 (5.50)	40		

*In 6 samples positive for GII norovirus, the Cq value could not be determined.

## Discussion

Our results show that 18.78% of AGE outbreaks occurred in LTCF, similar to the results obtained by Torner et al.^29^ in a study carried out in Catalonia in 2010 and 2011.

Norovirus was identified as the cause of 75% of the 40 outbreaks occurring in LTCF, coinciding with results from other studies. Inns et al. described 566 AGE outbreaks in LTCF in Northeast England between 2016 and 2018 and norovirus was detected in 64% of outbreaks with an identified pathogen^30^. Steele et al.^31^ studied 7094 norovirus outbreaks between 2009 and 2017 in the United States of which 5335 (75%) occurred in LTCF and Espenhain et al. found that 77% of norovirus outbreaks in Norway between 2005 and 2018 occurred in LTCF^32^.

A seasonal distribution was observed, with most outbreaks occurring in the cool months. Our results are consistent with previous findings by other authors indicating a seasonality of norovirus disease^4,33^.

The mode of transmission of the outbreaks studied showed there was person-to-person transmission in 90% and only 10% were foodborne. Similar results were found by Kroneman et al. in a study of norovirus outbreaks in 13 European countries between July 2001 and June 2006 (person-to-person transmission accounted for 88% of outbreaks, 10% were foodborne and 2% were food and waterborne)^34^. We found no outbreaks due to waterborne transmission. Chen et al. in a review of norovirus outbreaks in LTCF found person-to-person transmission in > 90% of outbreaks and linked this to close contact with other residents, shared facilities and contact with visitors and staff^35^. Lian et al.^4^ in an analysis of norovirus outbreaks reported in China from 2014 to 2017 found that 77% were caused by person-to-person transmission, 6% foodborne, 4% waterborne and 13% by multiple transmission.

Hospitalization was required in 1.08% of affected people, coinciding with Espenhaim et al.^32^ in a Norwegian study carried out during 2005–2018, who reported 0.91% of hospitalizations in affected people.

No deaths were reported in the norovirus outbreaks included in this study, coinciding with the results of the Lian et al. study carried out in China in a four-year period^4^. However, Espenhaim et al.^32^ reported 0.67% of deaths in LTCF outbreaks in the above-mentioned period of 13 years.

Our results suggest that the closeness of contact between residents and staff may play an important role in the transmission, as staff who had greater contact with residents (healthcare staff and caregivers) had an increased risk of being affected than those who did not. A 2014 meta-analysis by Petrignani et al.^36^ of 40 outbreaks in LTCF also found that the closeness of contact between staff and residents was related to the risk of staff being affected. The authors found that residents with medium or high dependence had a higher attack rate than those with low dependence. Our results showed that 70.21% of affected residents had total or severe dependence (Barthel score between 0 and 60) in agreement with the results obtained by these authors, suggesting that people with greater dependence require greater contact with caregivers.

Norovirus was identified by RTqPCR in the stool samples of 36.70% (40/109) of asymptomatic exposed persons, similar to the values estimated by Miura et al. in foodborne outbreaks in Japan from 2005 to 2006 in which they identified norovirus in 32.1% of asymptomatic persons^37^.

We found that symptomatic infected persons had a higher viral load, with a mean Cq of 25.65, compared with 29.00 in asymptomatic infected persons. These results are similar to those obtained by Shioda et al., who studied 12,910 samples from outbreaks and isolated cases in the United States and Latin America with a mean Cq of 25.3 for symptomatic affected persons and 28.5 for asymptomatic affected persons^18^.

We found that outbreaks in centers with a lower capacity had a higher attack rate than outbreaks in centers with greater capacity. A possible explanation is that, in smaller centers, the cleaning and disinfection protocols when outbreaks occur may be less developed than in large centers. Rosenthal et al. in a study conducted in Oregon between 2003 and 2006, found contrary results^38^, with the differences possibly being due to characteristics of the centers that were not recorded.

A strength of the study is that all outbreaks reported in a region with the same surveillance system were included, and therefore the results should be homogeneous and reflect the real situation.

Our study has some limitations. First, the mild severity of AGE outbreaks due to norovirus means underreporting may be greater in small centers than in large ones as, because there are fewer cases, contact with the health services and notification of the outbreak may be less likely. Secondly, we did not collect information about staff working closely with residents with different levels of dependence and, therefore, we could not analyze whether there was a relationship between these variables. Another limitation is that the number of samples from infected asymptomatic persons was low, meaning there was not sufficient statistical power to detect differences with symptomatic persons.

## Conclusions

Norovirus caused the vast majority of AGE outbreaks in LTCF, with more residents than staff being affected, especially those with a high degree of dependence. Person-to-person transmission was the main mode of transmission and GII was the most prevalent causal agent. There was no outbreak caused by GIV.

Mean viral loads were higher in infected symptomatic persons than in infected asymptomatic persons, both globally and in residents. Because norovirus was detected in asymptomatic persons, control measures should be applied not only to people with symptoms but to all persons in LTCF where norovirus outbreaks occur.

## Data Availability

The datasets generated during the current study are available in the Mendeley Data repository (https://data.mendeley.com/datasets/58pjx5vpk2/1).
